# Meningococcal Meningitis Complicated by Cerebral Venous Sinus Thrombosis Presenting as Pseudo-Subarachnoid Hemorrhage in a Toddler: A Case Report

**DOI:** 10.7759/cureus.104710

**Published:** 2026-03-05

**Authors:** Ahmad Altelly, Khulood Al-Dahash, Hussein Hamed, Mohamed Elkafafi, Rajesh Phatak

**Affiliations:** 1 Academic Affairs, Burjeel Hospital, Abu Dhabi, ARE; 2 Academic Affairs, Tawam Hospital, Al Ain, ARE; 3 Pediatric Intensive Care Unit, Burjeel Hospital, Abu Dhabi, ARE

**Keywords:** anticoagulation, bacterial meningitis complications, cerebral venous sinus thrombosis, dural sinus thrombosis, enoxaparin, neisseria meningitidis, neuroimaging pitfall, pediatric meningitis, pediatric stroke, pseudo-subarachnoid hemorrhage sign

## Abstract

Cerebral venous sinus thrombosis (CVST) is a rare and potentially devastating complication of bacterial meningitis. While a small number of adult cases have been reported, meningococcal-associated CVST in children is exceptionally rare. We report a previously healthy 20-month-old boy who presented with fever, vomiting, irritability, and a left convergent squint. Initial non-contrast computed tomography of the brain demonstrated bilateral tentorial hyperdensities consistent with the pseudo-subarachnoid hemorrhage sign, an imaging pitfall in which venous engorgement and cerebral edema mimic true subarachnoid hemorrhage. Subsequent magnetic resonance venography and computed tomography venography confirmed acute thrombosis of the right transverse and sigmoid sinuses. Cerebrospinal fluid polymerase chain reaction obtained on the third day of admission confirmed *Neisseria meningitidis*. The patient was treated with appropriate antimicrobial therapy, intensive supportive care, and therapeutic anticoagulation with enoxaparin, achieving progressive clinical improvement without hemorrhagic complications. Thrombophilia workup, including complement levels, immunoglobulins, protein C, protein S, and antithrombin III, was unremarkable. To the best of our knowledge, this represents the first reported case of meningococcal meningitis complicated by CVST in a child without an identified thrombophilic predisposition, suggesting that meningococcal infection alone may be sufficient to precipitate CVST. This case underscores the importance of maintaining a high index of suspicion for CVST, recognizing the pseudo-subarachnoid hemorrhage sign as a potential diagnostic pitfall, and pursuing early venographic imaging when neurological deterioration occurs despite appropriate antimicrobial therapy.

## Introduction

Acute bacterial meningitis remains a significant cause of morbidity and mortality among children worldwide [1]. While complications such as seizures, hearing loss, and hydrocephalus are well-documented, vascular complications, particularly cerebral venous sinus thrombosis (CVST), are far less frequent and carry a high risk of neurological deterioration and long-term disability [1,2].

In adults, CVST as a complication of bacterial meningitis is rare, occurring in less than 1% of cases, with *Streptococcus pneumoniae* accounting for the majority (65%) of identified pathogens [3]. Pediatric data remain scarce, with only a limited number of reports describing CVST complicating bacterial meningitis in children [2,4,5]. In a recent pediatric cohort, Mehta et al. (2025) identified CVST in 20 of 67 children presenting with meningitis; notably, none of these cases were attributable to *Neisseria meningitidis* [2].

CVST complicating meningococcal meningitis has been described in adults in only a small number of case reports [6,7]. In the pediatric population, a single case report documented CVST following meningococcal meningitis in an eight-month-old infant subsequently found to have hyperhomocysteinemia and methylenetetrahydrofolate reductase (MTHFR) variants (C677T and A1298C) [4]. Notably, no pediatric case of meningococcal-associated CVST has been reported in the absence of an underlying thrombophilic disorder.

Diagnosis of CVST in the setting of acute meningitis is particularly challenging, as clinical manifestations frequently overlap with those of meningitis itself [8]. Early non-contrast computed tomography (CT) can be normal or demonstrate nonspecific findings [3]. The pseudo-subarachnoid hemorrhage sign, in which diffuse cerebral edema or engorged venous structures produce increased cisternal attenuation mimicking true subarachnoid hemorrhage on CT, may result in diagnostic misinterpretation and delay [9,10]. Importantly, CVST itself is a recognized direct cause of this sign through engorgement of thrombosed dural sinuses, creating a compounded diagnostic challenge when both conditions coexist.

To the best of our knowledge, this represents the first reported case of meningococcal meningitis complicated by CVST in a child without an underlying thrombophilic predisposition, illustrating two underrecognized challenges: the exceptional rarity of this pathogen-complication association in children, and the pseudo-subarachnoid hemorrhage sign as a compounding diagnostic pitfall.

## Case presentation

A previously healthy 20-month-old boy was referred to our emergency department from an outside facility with suspected meningitis. Three days prior to presentation, he developed a persistent fever unresponsive to antipyretics, associated with non-bilious vomiting. At the referring facility, he was noted to be irritable with a left convergent squint, prompting urgent transfer.

There was no history of abdominal pain, diarrhea, constipation, recent travel, or sick contacts. The patient had an unremarkable birth history with age-appropriate developmental milestones. He had no prior medical or surgical history, no known allergies, and was not on any regular medications. Immunizations were up to date according to the United Arab Emirates (UAE) national vaccination guidelines.

On arrival, the patient appeared acutely unwell, irritable, and febrile. His vital signs were as follows: temperature of 38.1°C (tympanic), heart rate of 170 beats per minute, respiratory rate of 30 breaths per minute, and blood pressure of 102/54 mmHg. Neurological examination revealed depressed consciousness with marked irritability and a Pediatric Glasgow Coma Scale score of 9/15 (E2V2M5). Pupils were equal and reactive, and a left convergent squint was noted; detailed assessment of extraocular movements was limited by reduced consciousness. Neck rigidity was marked with a positive Brudzinski sign. Examination of other systems was unremarkable.

The patient was admitted to the pediatric intensive care unit (PICU). Initial management included intravenous 3% hypertonic saline, empirical ceftriaxone, vancomycin, and adjunctive dexamethasone. Levetiracetam was additionally initiated for seizure prophylaxis.

**Figure 1:**
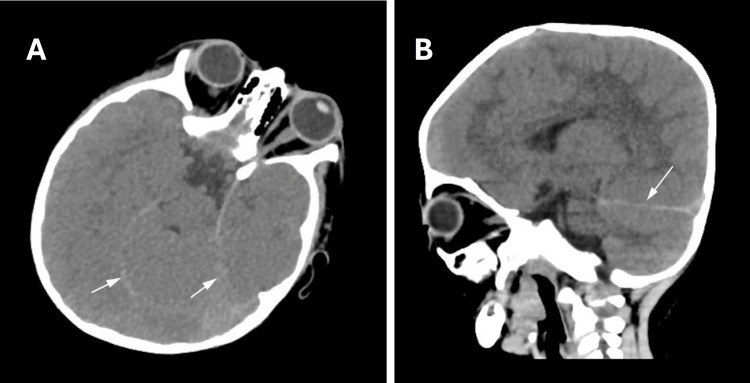
Non-contrast computed tomography of the brain on admission. (A) Axial image demonstrating bilateral linear hyperdensities along the tentorial margins (white arrows). (B) Sagittal reconstruction demonstrating corresponding tentorial hyperdensity (white arrow). No parenchymal hemorrhage, mass effect, midline shift, or hydrocephalus was identified. These appearances initially raised concern for subarachnoid hemorrhage and prompted advanced venographic imaging.

Laboratory investigations demonstrated markedly elevated C-reactive protein (CRP) and procalcitonin, leukocytosis with neutrophil predominance, metabolic acidosis, and mild hepatic dysfunction (Table 1). Blood and urine cultures, respiratory viral panel, and SARS-CoV-2 PCR were negative. Admission non-contrast CT of the brain revealed bilateral linear hyperdensities along the tentorial margins (Figure 1). No parenchymal hemorrhage, mass effect, midline shift, or hydrocephalus was identified.

**Table 1:** Laboratory investigations at admission and during hospital course. ↑, above reference range; ↓, below reference range; —, not performed or not applicable; ALP, alkaline phosphatase; ALT, alanine aminotransferase; aPTT, activated partial thromboplastin time; AST, aspartate aminotransferase; CSF, cerebrospinal fluid; IgA/G/M/E, immunoglobulins; INR, international normalized ratio; LDH, lactate dehydrogenase; MRSA, methicillin-resistant *Staphylococcus aureus*; PCR, polymerase chain reaction; PT, prothrombin time; WBC, white blood cell count.

Parameter	Day 1 (Admission)	Day 3	Day 4	Day 9 (Pre-discharge)	Reference range
Inflammatory markers					
C-reactive protein (mg/L)	341 ↑	—	136 ↑	3.0	<5
Procalcitonin (ng/mL)	100 ↑	84.7 ↑	37.2 ↑	1.49 ↑	<0.05
Complete blood count					
White blood cell count (×10⁹/L)	15.22 ↑	—	10.92	11.63	4.0-11.0
Neutrophils (%)	74	—	47	64	40-75
Neutrophil absolute (×10⁹/L)	11.22 ↑	—	5.09	7.48 ↑	1.5-7.5
Hemoglobin (g/dL)	10.0 ↓	—	8.3 ↓	10.2 ↓	12.0-16.0
Platelets (×10⁹/L)	196 ↓	—	178 ↓	590 ↑	150-400
Cerebrospinal fluid analysis (day 3)					
Appearance	—	Turbid	—	—	Clear
WBC count (cells/μL)	—	208 ↑	—	—	0-5
Polymorphs (%)	—	58	—	—	0-6
Predominant cell type	—	Neutrophils	—	—	None
Protein (g/L)	—	0.737 ↑	—	—	0.15-0.45
Glucose (mmol/L)	—	5.22 ↑	—	—	2.5-4.4
LDH (U/L)	—	324 ↑	—	—	< 40
PCR: *N. meningitidis*	—	Detected	—	—	Not detected
Liver function					
ALT (U/L)	62 ↑	—	67 ↑	—	7-56
AST (U/L)	41 ↑	—	35	—	10-40
ALP (U/L)	158 ↑	—	133 ↑	—	40-130
Albumin (g/L)	35.4 ↓	—	32.2 ↓	—	35-50
Bilirubin, total (μmol/L)	12.9	—	2.7	—	5-21
Bilirubin, direct (μmol/L)	7.8 ↑	—	—	—	0-7
LDH (U/L)	359 ↑	—	—	—	140-280
Coagulation					
PT (seconds)	14.90 ↑	—	9.70	—	11.0-13.5
INR	1.4 ↑	—	0.9	—	0.8-1.2
aPTT (seconds)	33.8	—	27.6	—	25-35
Thrombophilia and immunology (pre-discharge)					
Protein C activity (%)	—	—	136	—	70-140
Protein S activity (%)	—	—	106	—	60-130
Antithrombin III activity (%)	—	—	120	—	80-120
C3 complement (g/L)	—	—	1.8	—	0.9-1.8
C4 complement (g/L)	—	—	0.3	—	0.1-0.4
IgA (g/L)	—	0.47	—	—	0.7-4.0
IgG (g/L)	—	7.43	—	—	7.0-16.0
IgM (g/L)	—	0.94	—	—	0.4-2.3
Total IgE (IU/mL)	—	51.9	—	—	<100
Microbiology					
Blood culture	Negative	—	—	—	Negative
CSF culture	—	Negative	—	—	Negative
SARS-CoV-2 PCR	Negative	—	—	—	Negative
Respiratory viral panel	Negative	—	—	—	Negative
MRSA screen	Negative	—	—	—	Negative

Approximately four hours after PICU admission, neurological status deteriorated, necessitating endotracheal intubation and mechanical ventilation. Due to instability, lumbar puncture was deferred at this time. Magnetic resonance imaging (MRI) of the brain with and without contrast, including magnetic resonance angiography (MRA) and magnetic resonance venography (MRV), was requested. Fundoscopic examination revealed no papilledema.

**Figure 2:**
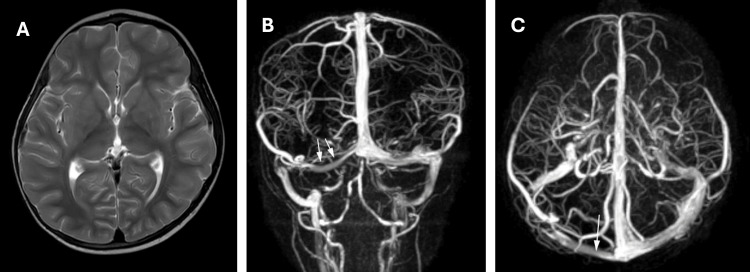
Brain magnetic resonance imaging with magnetic resonance venography on the second day of admission. (A) Axial T2-weighted image demonstrating fluid-fluid levels within the occipital horns of both lateral ventricles with dependent layering, consistent with proteinaceous or turbid cerebrospinal fluid rather than intraventricular hemorrhage. (B) Coronal magnetic resonance venography demonstrating irregular opacification of the right transverse and sigmoid sinuses (white arrows), consistent with venous sinus thrombosis. (C) Sagittal magnetic resonance venography demonstrating a linear filling defect with nodularity along the lateral aspect of the right transverse sinus (white arrow), consistent with acute dural venous sinus thrombosis.

MRI on the second day demonstrated no leptomeningeal enhancement or parenchymal signal abnormality. Axial T2-weighted imaging demonstrated fluid-fluid levels within the occipital horns bilaterally (Figure 2A). A linear filling defect with nodularity along the right transverse sinus was identified on MRV (Figures 2B, 2C). MRA was normal.

**Figure 3:**
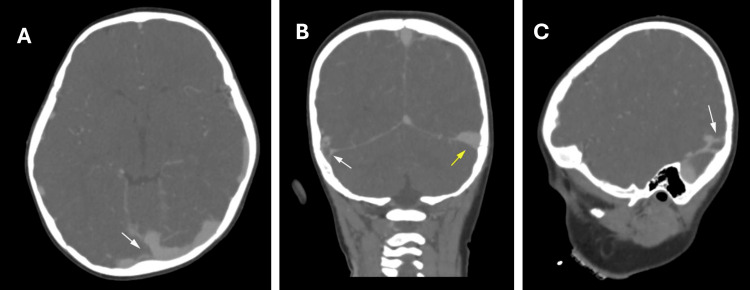
Contrast-enhanced computed tomography venography on the fourth day of admission. (A) Axial image demonstrating reduced contrast opacification of the right transverse-sigmoid sinus region compared with the left side (white arrow). (B) Coronal image demonstrating non-opacification of the right transverse sinus (white arrow) with preserved opacification of the left transverse sinus (yellow arrow), consistent with acute dural venous sinus thrombosis. (C) Sagittal reconstruction demonstrating non-opacification of the right transverse and sigmoid sinuses (white arrow).

Lumbar puncture on the third day revealed slightly turbid cerebrospinal fluid (CSF) with neutrophilic pleocytosis. CSF polymerase chain reaction (PCR) was positive for *Neisseria meningitidis*. CT angiography (CTA) and CT venography (CTV) performed on the fourth day demonstrated reduced opacification of the right transverse-sigmoid sinus on CTA (Figure 3A), and non-opacification of the right transverse and sigmoid sinuses with nodular tentorial enhancement on CTV (Figures 3B, 3C). Therapeutic enoxaparin 1 mg/kg twice daily was initiated.

**Figure 4:**
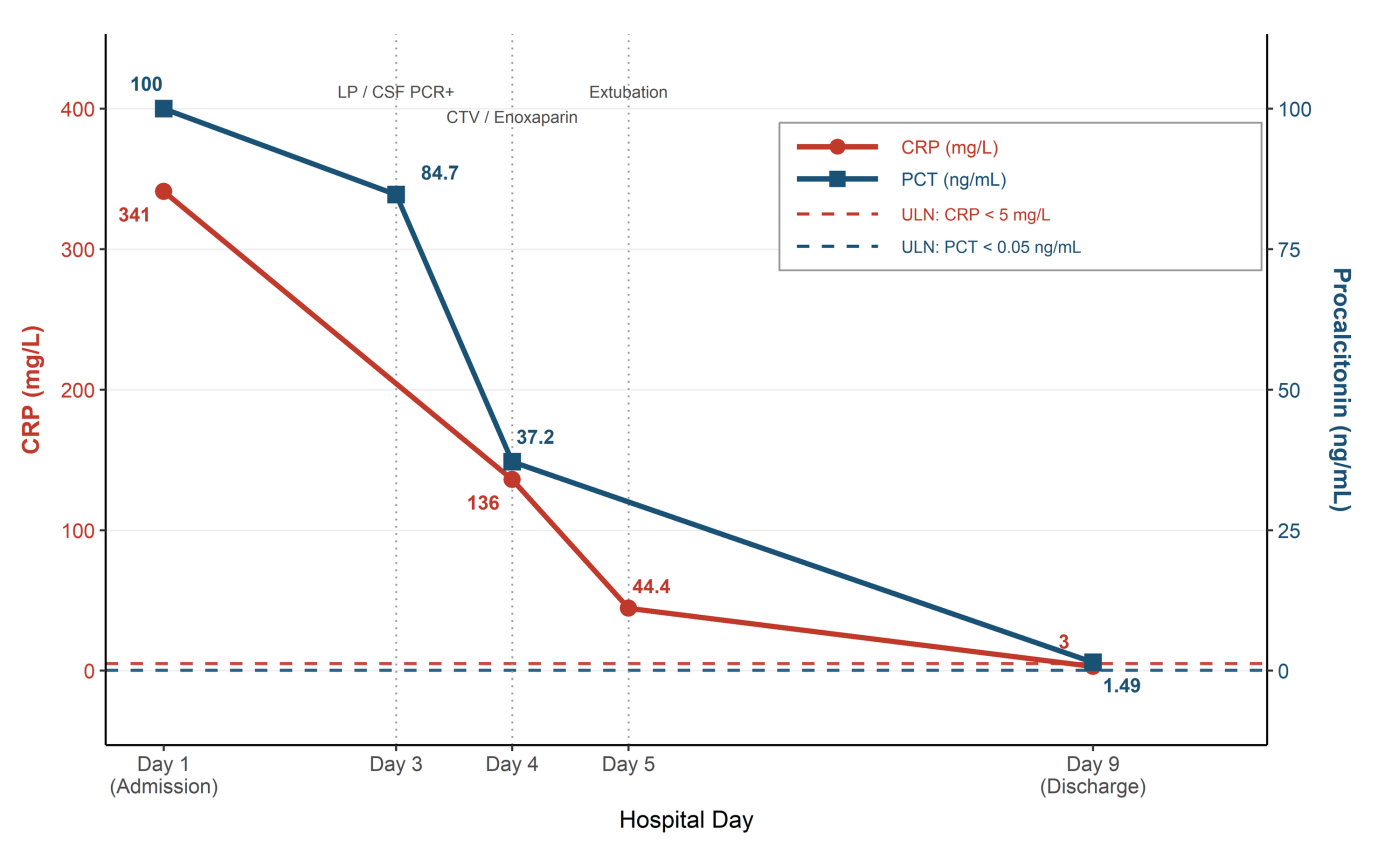
Trend of inflammatory markers during the hospital course. Serial C-reactive protein (CRP; red circles, left y-axis, mg/L) and procalcitonin (PCT; blue squares, right y-axis, ng/mL) values are plotted across the nine-day hospital course. Both markers were markedly elevated at admission (CRP = 341 mg/L; PCT = 100 ng/mL) and declined steadily, reaching near-normal levels by discharge (CRP = 3 mg/L; PCT = 1.49 ng/mL). Dashed color-coded horizontal lines denote the upper limit of normal (ULN) for each marker (CRP < 5 mg/L; PCT < 0.05 ng/mL). Vertical dotted lines indicate key clinical events: lumbar puncture (LP) with positive cerebrospinal fluid (CSF) polymerase chain reaction (PCR) on day three, computed tomography venography (CTV) and initiation of enoxaparin on day four, and extubation on day five.

By the fourth day, inflammatory markers showed a significant decline (Table 1 and Figure 4). By the fifth day, levetiracetam and vancomycin were discontinued, and the patient was successfully extubated and later shifted to the ward on the sixth day. Ceftriaxone was completed on the eighth day. The enoxaparin regimen was consolidated to 2 mg/kg once daily prior to discharge. On the ninth day, inflammatory markers showed marked improvement, and the white blood cell count was within normal limits (Table 1 and Figure 4).

Prior to discharge, complement levels (CH50, C3, C4), immunoglobulin levels, protein C, protein S, and antithrombin III were within normal limits (Table 1). The patient was discharged on the tenth day with anticoagulation planned for three months and follow-up MRI with MRA and MRV scheduled for three to six months post-discharge. At one-week neurology follow-up, the family reported continued clinical improvement.

## Discussion

CVST is an uncommon but clinically significant complication of community-acquired bacterial meningitis, carrying a risk of venous infarction, intracranial hemorrhage, and raised intracranial pressure if not promptly recognized [3]. In the largest prospective cohort evaluating CVST in adult bacterial meningitis, CVST occurred in approximately 1% of cases and was associated with higher rates of coma, focal deficits, unfavorable outcomes, and mortality [3]. Pediatric data remain considerably more limited [2,4,5].

*Streptococcus pneumoniae *accounts for the majority of adult cases of CVST complicating bacterial meningitis, whereas* Neisseria meningitidis *is only infrequently implicated [3]. In the Mehta et al. (2025) cohort, *Neisseria meningitidis *was not isolated in any of the 20 pediatric CVST cases identified [2]. The only prior pediatric case involved an infant with an underlying thrombophilic disorder [4]. Our patient had no identifiable thrombophilia or immunodeficiency on basic screening, suggesting that meningococcal infection alone may be sufficient to precipitate CVST in young children.

The pathophysiological basis for this can be conceptualized through Virchow's triad [3,8]. *Neisseria meningitidis* produces direct endothelial injury through bacterial invasion and lipooligosaccharide release, activating the coagulation cascade via tissue factor expression. Concurrent venous stasis results from raised intracranial pressure, cerebral edema, and dehydration. The systemic septic response further induces a hypercoagulable state through consumption of protein C, protein S, and antithrombin III. All three components were plausibly present in our patient without requiring a hereditary predisposition. Observational registries further support a multifactorial model in which infection and physiological stressors combine to precipitate thrombosis [11,12].

The left convergent squint noted at presentation warrants comment. In the context of raised intracranial pressure or impaired venous outflow from dural sinus thrombosis, abducens nerve palsy is a well-recognized localizing sign resulting from stretching of the sixth cranial nerve [8]. Its presence alongside neurological deterioration appropriately heightened clinical concern and contributed to the decision to pursue advanced neuroimaging.

Diagnosis of CVST during acute meningitis presents compounding clinical and radiological challenges. Clinically, altered mental status and focal neurological deficits overlap substantially with meningitis itself [2,3]. Radiologically, early non-contrast CT may be normal or nonspecific [3]. In our case, the admission non-contrast CT demonstrated bilateral tentorial hyperdensities initially interpreted as possible subarachnoid hemorrhage, leading to lumbar puncture deferral. These appearances were consistent with the pseudo-subarachnoid hemorrhage sign, in which diffuse cerebral edema and engorged venous structures produce increased cisternal and tentorial attenuation mimicking true subarachnoid blood on non-contrast CT [9,10,13,14]. Critically, CVST itself directly causes this sign through engorgement of thrombosed dural sinuses, meaning both meningitis-related edema and the CVST likely contributed simultaneously in our patient. The bilateral distribution of hyperdensities, in the context of a right-sided thrombosis, further supports a diffuse edema-related rather than focal hemorrhagic mechanism. Early venographic imaging with CTV or MRV is therefore essential when CVST is suspected in patients with meningitis and evolving neurological findings [2,3]. Confirmatory CTV was performed prior to anticoagulation, given the recognized risk of false-positive MRV findings and the importance of unequivocal confirmation before anticoagulating in active central nervous system (CNS) infection.

Management of pediatric CVST is guided primarily by observational data. Anticoagulation with low-molecular-weight heparin has been shown to be safe in pediatric cohorts, with evidence supporting reduced thrombus propagation and acceptable bleeding risk [11,15]. Registry data from the International Pediatric Stroke Study associate anticoagulation with improved short-term outcomes in childhood CVST [12]. In our patient, therapeutic enoxaparin was initiated following CTV confirmation and continued for three months post discharge, with no hemorrhagic complications.

Published epidemiological data from the United Arab Emirates are limited; however, regional data from Al Ain demonstrate a persistent burden of pediatric meningitis, particularly among young children, supporting the local relevance of recognizing rare but serious complications such as CVST [16]. This case contributes to the sparse regional and global pediatric literature on meningococcal-associated CVST and highlights the need for heightened clinical vigilance, early recognition of radiological pitfalls, and prompt venographic imaging when clinical deterioration occurs.

This report has three limitations. First, as a single case report, causality between meningococcal infection and CVST cannot be definitively established. Second, the thrombophilic workup was not comprehensive. MTHFR mutation analysis, homocysteine levels, prothrombin gene mutations, and testing for hemoglobinopathies were not obtained, and are recommended in future similar cases. Furthermore, protein C, protein S, and antithrombin III levels measured during the acute phase may be unreliable due to consumption coagulopathy, and repeat outpatient testing at three months post recovery is recommended. Third, long-term neurodevelopmental outcomes and venographic confirmation of recanalization were not available at the time of writing.

## Conclusions

CVST is a rare but serious complication of meningococcal meningitis in children, and its diagnosis may be delayed by overlapping clinical features and misleading neuroimaging. The pseudo-subarachnoid hemorrhage sign represents an underrecognized radiological pitfall that can delay lumbar puncture and venographic imaging. Early recourse to MRV or CTV when neurological deterioration occurs despite appropriate antimicrobial therapy is essential. Future cases should include a comprehensive thrombophilic evaluation to investigate any underlying thrombophilic etiology.
